# Ruthenium Complexes Induce HepG2 Human Hepatocellular Carcinoma Cell Apoptosis and Inhibit Cell Migration and Invasion through Regulation of the Nrf2 Pathway

**DOI:** 10.3390/ijms17050775

**Published:** 2016-05-19

**Authors:** Yiyu Lu, Ting Shen, Hua Yang, Weiguang Gu

**Affiliations:** Department of Oncology, Nanhai Hospital of Southern Medical University, Foshan 528200, China; lu_yiyu@163.com (Y.L.); shenting0479@163.com (T.S.); yanghua9206@163.com (H.Y.)

**Keywords:** Ru complex, apoptosis, Nrf2

## Abstract

Ruthenium (Ru) complexes are currently the focus of substantial interest because of their potential application as chemotherapeutic agents with broad anticancer activities. This study investigated the *in vitro* and *in vivo* anticancer activities and mechanisms of two Ru complexes—2,3,7,8,12,13,17,18-Octaethyl-21H,23H-porphine Ru(II) carbonyl (**Ru1**) and 5,10,15,20-Tetraphenyl-21H,23H-porphine Ru(II) carbonyl (**Ru2**)—against human hepatocellular carcinoma (HCC) cells. These Ru complexes effectively inhibited the cellular growth of three human hepatocellular carcinoma (HCC) cells, with IC_50_ values ranging from 2.7–7.3 μM. In contrast, the complexes exhibited lower toxicity towards L02 human liver normal cells with IC_50_ values of 20.4 and 24.8 μM, respectively. Moreover, **Ru2** significantly inhibited HepG2 cell migration and invasion, and these effects were dose-dependent. The mechanistic studies demonstrated that **Ru2** induced HCC cell apoptosis, as evidenced by DNA fragmentation and nuclear condensation, which was predominately triggered via caspase family member activation. Furthermore, HCC cell treatment significantly decreased the expression levels of Nrf2 and its downstream effectors, NAD(P)H: quinone oxidoreductase 1 (NQO1) and heme oxygenase 1 (HO1). **Ru2** also exhibited potent *in vivo* anticancer efficacy in a tumor-bearing nude mouse model, as demonstrated by a time- and dose-dependent inhibition on tumor growth. The results demonstrate the therapeutic potential of Ru complexes against HCC via Nrf2 pathway regulation.

## 1. Introduction

The success of cisplatin in combating cancers has kindled substantial interest among scientists to identify additional, platinum (Pt)-based metal complexes with improved anticancer activities [1,2]. However, clinically serious side effects, such as nephrotoxicity, gastrointestinal toxicity, and drug resistance, have limited the clinical applications of Pt-based complexes [3]. Therefore, substantial efforts have been made in previous decades to identify non-platinum-based metal anticancer drugs [4,5,6,7]. Specifically, ruthenium (Ru)-based complexes comprise promising antitumor agents with broad anticancer effects. These Ru-based complexes may function as alternatives to cisplatin and platinum-based anticancer drugs because they exhibit favorable properties for the design of anticancer drugs, such as the higher coordination number for more diversified drug design and lower toxicity with respect to healthy tissues [8,9,10,11].

Human hepatocellular carcinoma (HCC), which accounts for approximately 700,000 yearly deaths worldwide, is the fifth and seventh most common cancer in men and women, respectively [12]. Currently, chemotherapy is one of the most important modalities for HCC treatment. Therefore, the rational design and synthesis of potent metal complexes, especially Ru complexes, may represent a potential strategy to identify novel anti-HCC drugs. Studies have demonstrated the application potentials of Ru complexes in the chemotherapy of HCC. For example, Chen and colleagues demonstrated that water-soluble Ru(II) complexes with chiral 4-(2,3-dihydroxypropyl)-formamide oxoaporphine functioned as G4-DNA binders to inhibit telomerase activity and subsequently induce HCC cell apoptosis [13]. Yuan *et al.* demonstrated that the Ru complex Λ-WH0402 induced HCC LM6 cell death by triggering Beclin-1-dependent autophagy pathways [14]. Wu *et al.* demonstrated that a structural change in the Ru(II) arene complex from a ferrocene unit to a carboxyl group led to highly selective antitumor activity aimed at cancerous cells and facilitated the inhibition of cancer cell proliferation [15]. Moreover, Huang *et al.* determined that mixed-ligand Ru(II) complexes could be used for photodynamic therapy of HCC cells [16]. Taken together, these findings indicate the therapeutic potential of Ru complexes for HCC treatment. However, the *in vivo* effects and mechanisms remain unclear.

The current study investigated the anti-HCC activities and mechanisms of Ru complexes *in vitro* and *in vivo*. The findings indicated that Ru complexes inhibited HCC cell growth, migration, and invasion via the induction of Nrf2-mediated cell apoptosis. Furthermore, Ru complex 2 (**Ru2**) demonstrated potent *in vivo* anti-HCC activity in an animal model. Taken together, the current findings support the therapeutic potential of Ru complexes against HCC via Nrf2 pathway regulation.

## 2. Results and Discussion

### 2.1. Ru Complexes Inhibit HCC Cell Growth, Migration, and Invasion

**Figure 1:**
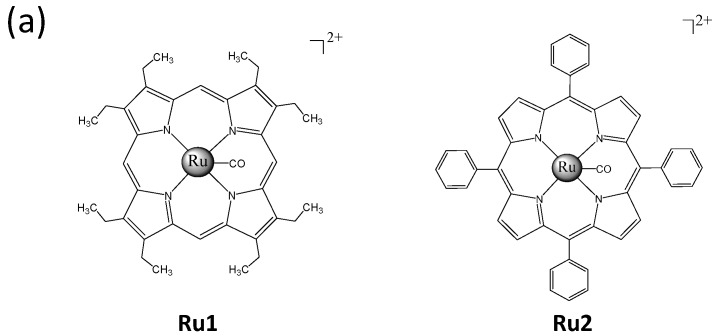

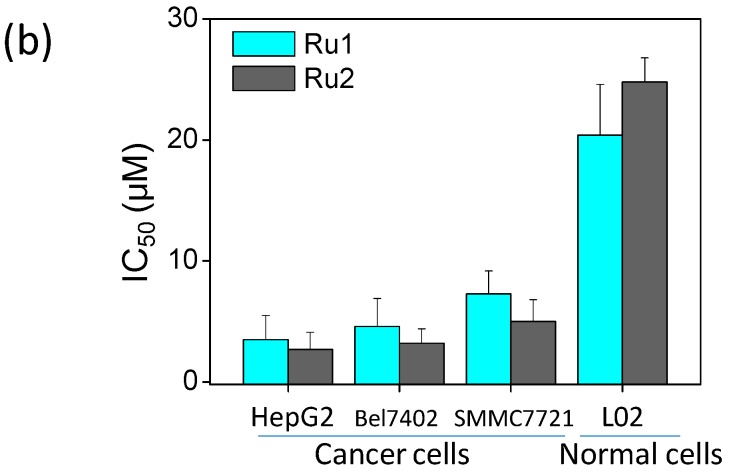
Ru complexes exert *in vitro* anticancer activities. (**a**) Chemical structure of Ru complexes; (**b**) Cytotoxicity of Ru complexes towards human cancer and normal cells. Human hepatocellular carcinoma (HCC) and L02 cells were treated with Ru complexes for 72 h. The cell viability was subsequently investigated via an MTT assay.

**Figure 2:**
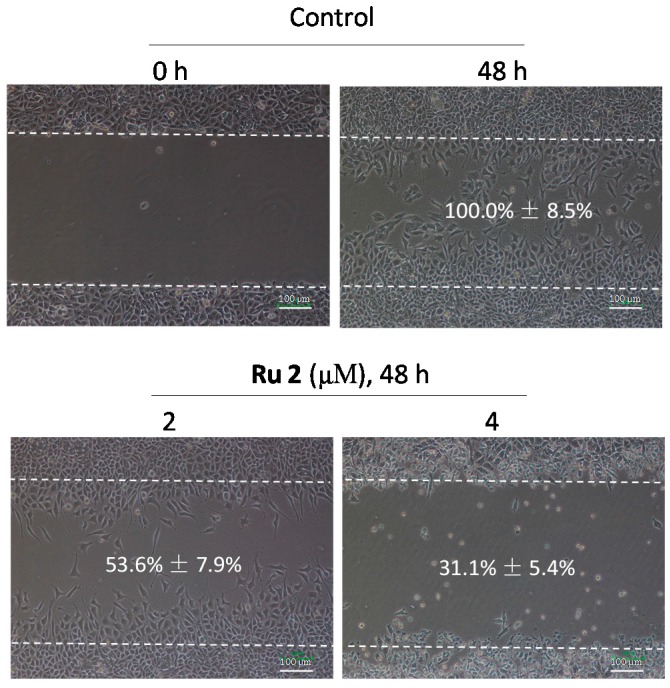
Effects of **Ru2** on HepG2 cell migration. Cells were exposed to different **Ru2** concentrations for 24 h and photographed using a phase-contrast microscope (200×, Nikon TS100, Nikon, Tokyo, Japan). Values in the images indicate the migration ability of the cells.

**Figure 3:**
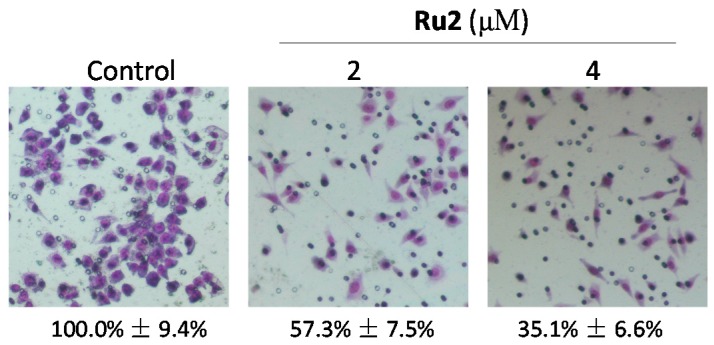
Effects of **Ru2** on HepG2 cell invasion by using Transwell Boyden assay. Cells were exposed to different **Ru2** concentrations for 24 h, stained with Giemsa solution, and photographed using a phase-contrast microscope (200×, Nikon TS100). Values under the images indicate the invasion ability of the cells.

The *in vitro* anticancer activities of two Ru complexes (Figure 1a) were first screened using several HCC cells (HepG2, Bel7402, and SMMC7721) and a human normal liver cell line (L02). Following 72 h of Ru complex treatment, the cell viability was assessed via an MTT assay. The IC_50_ value indicated that both **Ru1** and **Ru2** complexes could effectively inhibit HCC cell growth, with higher anticancer efficacy observed in **Ru2**, which exhibited IC_50_ values at 2.7 ± 1.4, 3.2 ± 1.2, and 5.0 ± 1.8 μM for the tested HCC cells (Figure 1b). This cell line-specific anticancer efficacy could be due to the different biochemical characteristic of different cells. In contrast, **Ru2** exhibited lower toxicity towards the L02 human normal liver cells (IC_50_ value = 24.8 ± 2.0 μM), which indicates a high selectivity between cancer and normal cells. As a result of the substantial metastatic potency of HCC, we investigated the effects of **Ru2** on the metastatic potential of HepG2 cells *in vitro*. A scratch motility assay was subsequently performed to determine the anti-metastatic potential of **Ru2**, and the results showed that **Ru2** at 2 and 4 μM effectively inhibited HepG2 cell migration following 24 h of treatment in a dose-dependent manner (Figure 2). Moreover, the effects of Ru complexes on the invasion of HepG2 cells were assessed using a Transwell Boyden chamber that was pre-coated with matrigel for 4 h at 37 °C. The results showed that **Ru2** effectively inhibited HepG2 cell invasion following 24 h of treatment in a dose-dependent manner (Figure 3). Together, these findings support the wide-spectrum therapeutic potential of Ru complexes in HCC treatment.

### 2.2. Cell Apoptosis Activation by Ru Complexes

**Figure 4:**
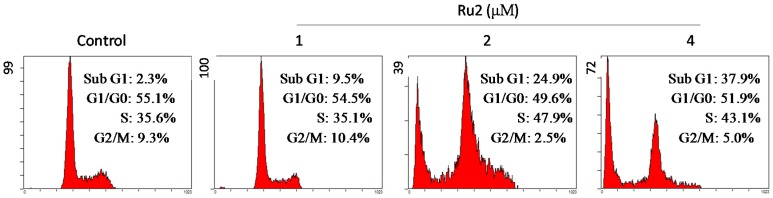
Quantitative analysis of the cell cycle distribution and apoptotic cell death as examined via flow cytometry. Cells were pretreated with or without **Ru2** for 24 h.

**Figure 5:**
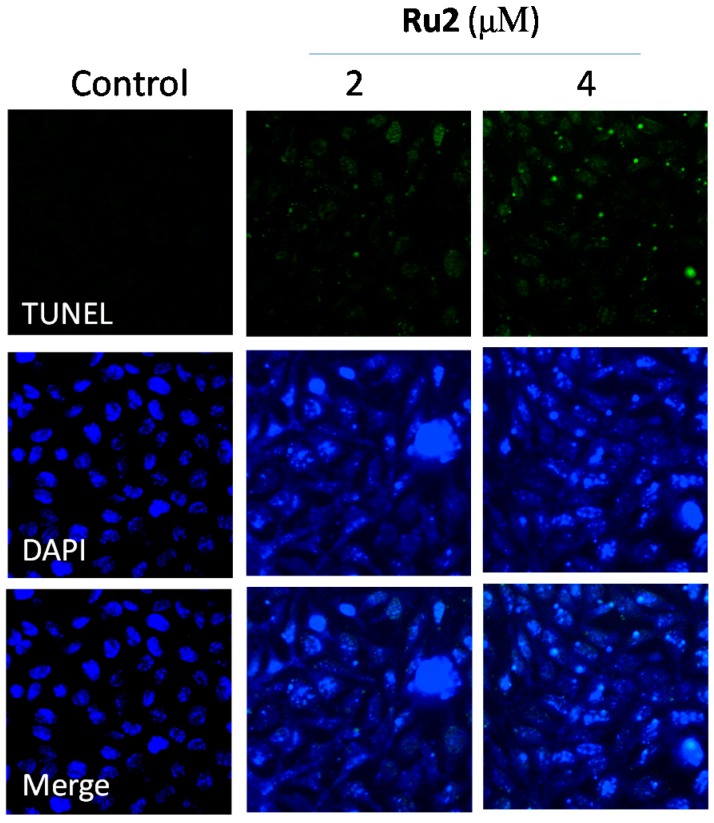
Apoptotic DNA fragmentation and nuclear condensation induced by **Ru2** as determined via a TUNEL-DAPI co-staining assay (magnification, 200×). Cells were treated with **Ru2** for 24 h.

Dysregulation of cell apoptosis has been associated with chronic diseases, especially cancers, in humans [17]. The induction of cancer cell apoptosis (also referred to as programmed cell death) has been demonstrated as an effective approach for clinical cancer treatment [18]. The growth inhibitory activities of most anticancer drugs on cancer cells are achieved via the induction of apoptosis [19]. Substantial evidence has demonstrated that Ru complexes inhibited cancer cell growth via the induction of apoptosis [5,9,11,16]. Based on *in vitro* anticancer screening, **Ru2** demonstrated an increased anticancer efficacy compared with **Ru1**. Thus, studies were conducted to investigate the anticancer mechanism through which **Ru2** caused cancer cell death. First, propidium iodide (PI)-flow cytometric analysis was implemented to identify the mechanism of Ru-induced cell death. HepG2 cell exposure to different **Ru2** concentrations led to an increased proportion of apoptotic cells (Figure 4); this effect was dose-dependent, as reflected by the increase in the sub-G1 populations from 2.3% (control) to 37.9% (4 μM) (Figure 4). Furthermore, the TUNEL-DAPI co-staining assay results indicated that HepG2 cell exposure to 2 and 4 μM of **Ru2** for 24 h triggered significant and dose-dependent apoptosis (Figure 5). Representative fluorescent images indicated that HepG2 cells treated with **Ru2** exhibited significant apoptotic DNA fragmentation and nuclear condensation. Overall, these findings indicate that apoptosis comprised the major mode of cell death induced by these Ru complexes.

**Figure 6:**
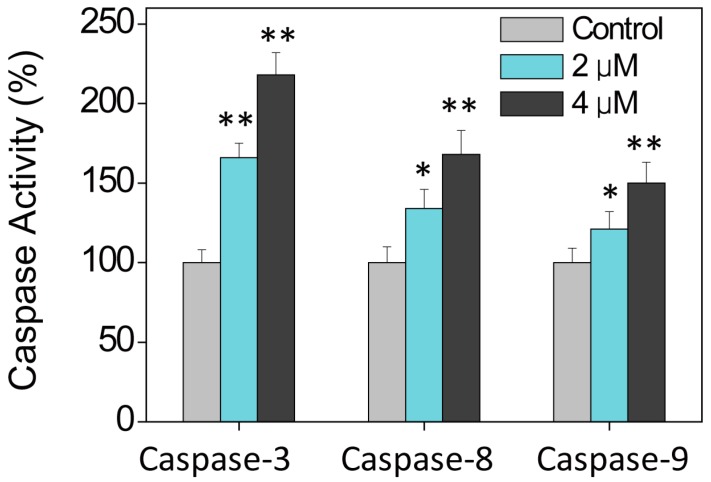
Caspase activities in HCC cells following **Ru2** exposure as evaluated via a specific fluorogenic substrate. * *p* < 0.05 and ** *p* < 0.01 compared with the control. Cells were treated with Ru complexes for 24 h.

Caspase family proteins function as important regulators in the induction of apoptosis via the enzymolysis of substrates [20]. Activated caspases induce proteolytic cleavage of PARP and ultimately result in cell apoptosis [20]. Cell apoptosis may be initiated by two mechanisms, including the death receptor-mediated extrinsic and mitochondria-mediated intrinsic apoptotic pathways. The current study investigated the intracellular caspase activities in cells following **Ru2** exposure to determine the caspase requirement for the apoptotic cascade. As shown in Figure 6, HepG2 cell exposure to **Ru2** significantly induced caspase-3, -8, and -9 activation. These findings indicate that **Ru2** induced HCC cell apoptosis primarily through the activation of caspase-mediated apoptosis, as well as the involvement of mitochondria and death receptors.

### 2.3. Ru Complexes Induce Reactive Oxygen Species (ROS) Overproduction via Nrf2 Pathway Regulation

**Figure 7:**
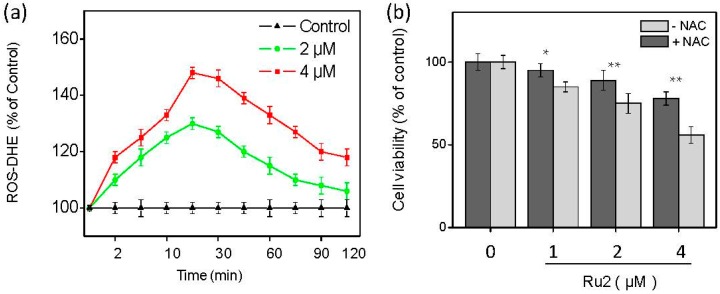
Important roles of ROS in cell apoptosis induced by **Ru2**. (**a**) Effects of concentration on intracellular ROS generation following **Ru2** treatment in HepG2 cells. The cells were treated with 10 µM DHE probe for 30 min; (**b**) Protective effects of *N*-acetylcysteine (NAC) on **Ru2**-induced growth inhibition. Bars with different characters are statistically different at * *p* < 0.05 and ** *p* < 0.01 levels. The cells were pretreated with NAC (1 mM) for 2 h followed by co-incubation with different concentrations of **Ru2**.

**Figure 8:**
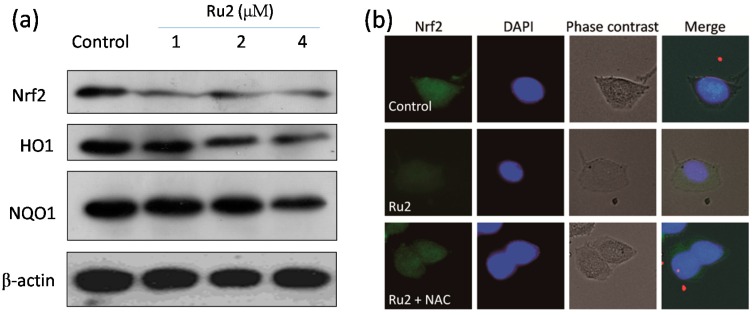
Regulation of Nrf2 pathway by **Ru2**. (**a**) Effects of **Ru2** on the expression levels of Nrf2, HO1, and NQO1 in HepG2 cells after treatments for 24 h; (**b**) Immuno-fluorescence of Nrf2 in cells treated with **Ru2** for 24 h (magnification, 200×).

ROS, including superoxide anion, hydrogen peroxide, and hydroxyl radical, are involved in the mechanisms of numerous anticancer drugs via the initiation of apoptotic signaling pathways during chemotherapy [21]. Excess intracellular ROS may cause DNA damage and trigger the activation of nuclear factor-like 2 (Nrf2) and downstream signals, such as NAD(P)H: quinone oxidoreductase 1 (NQO1) and heme oxygenase 1 (HO1) [22,23,24,25]. Substantial evidence indicates that ROS played a critical role in the signaling pathways triggered by therapeutic cancer drugs and metal complexes [5,9,11,16,26,27,28,29,30]. Therefore, we determined the intracellular ROS generation levels in HepG2 cells following **Ru2** exposure via the assessment of dihydroethidium (DHE) fluorescence intensity. The cells treated with **Ru2** significantly triggered ROS generation in a time- and dose-dependent manner (Figure 7a). Furthermore, when cells were treated with 4 µM of **Ru2**, the ROS production increased to 150% of the control. Moreover, the ROS scavenger N-acetylcysteine (NAC) was also implemented to confirm the role of ROS in cell apoptosis. These findings indicate that NAC pretreatment (1 mM) suppressed Ru-induced cell death (Figure 7b). Taken together, these findings indicate that ROS play an important role in cell apoptosis induced by Ru complexes in HCC cells.The Keap1-Nrf2 pathway is a critical cytoprotective regulator in mammalian cells in response to both endogenous and exogenous stresses [31]. Thus, Western blotting was used to identify the effects of **Ru2** on the Nrf2 expression level in HCC cells. **Ru2** significantly inhibited the Nrf2 signaling pathway, as demonstrated by the decreased expression levels of Nrf2 and its downstream effectors NQO1 and HO1, especially HO1, in a dose-dependent manner (Figure 8). These results suggest that the Nrf2 pathway may be involved in **Ru2**-induced oxidative stress and cell apoptosis. To clarify the involvement of the Nrf2 pathway in Ru-induced ROS generation and cell apoptosis, the effects of the ROS scavenger NAC on Nrf2 expression were investigated in cells with or without **Ru2** treatment. **Ru2** significantly reduced the Nrf2 protein level in the HCC cells (Figure 8), which was increased by the addition of NAC. These findings indicate that **Ru2** induces intracellular ROS generation to inhibit the Nrf2 signaling pathway to regulate cell fate.

### 2.4. In Vivo Anticancer Activities of **Ru2**

**Figure 9:**
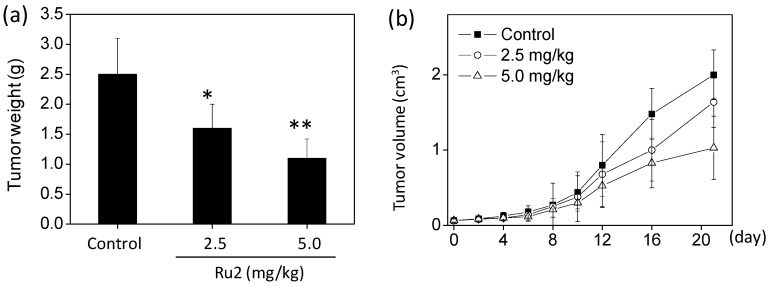
*In vivo* antitumor efficacy and action mechanisms of **Ru2**. Inhibition of HepG2 xenograft tumor weight (**a**) and tumor volume (**b**) by **Ru2**. BALB/c nude mice bearing HepG2 xenograft tumors were treated with the complex (2.5 and 5.0 mg/kg every other day) for 20 days. * *p* < 0.05 and ** *p* < 0.01 compared with the control.

Furthermore, the *in vivo* anticancer activity of **Ru2** against HCC was investigated in HepG2 xenografts using a nude mouse model. Following i.p. (2.5 and 5.0 mg/kg body weight on alternating days) administration for 20 days, the tumor weight significantly decreased from 2.5 to 1.6 and 1.1 g, respectively. The tumor volume dramatically decreased to 65% and 42%, respectively, of the control group (Figure 9). Moreover, the mice that received **Ru2** treatment maintained normal body weight throughout the treatment process, and the mortality for the mice in different treatment groups was found at zero. These findings support the potent *in vivo* anticancer efficacy of **Ru2** against HCC. Additional pre-clinical studies should be conducted to promote the development of this type of Ru-based anticancer metal medicine.

## 3. Materials and Methods

### 3.1. Reagents, Cell Lines, and Cell Cultures

Ru complexes, including 2,3,7,8,12,13,17,18-Octaethyl-21H,23H-porphine ruthenium(II) carbonyl and 5,10,15,20-Tetraphenyl-21H,23H-porphine ruthenium(II) carbonyl, and all other reagents were obtained from Sigma-Aldrich (St. Louis, MO, USA). Human HCC cells (HepG2, Bel7402, and SMMC7721) and a human normal liver cell line (L02) were purchased from American Type Culture Collection (ATCC, Manassas, VA, USA) and GuangZhou Jennio Biotech Co., Ltd. (Guangzhou, China). The cell lines were cultured and maintained in RPMI 1640 or DMEM culture media, which contained 10% fetal bovine serum, penicillin (100 units/mL), and streptomycin (50 units/mL) at 37 °C in a humid incubator with 5% CO_2_.

### 3.2. Cell Viability Assessment via MTT Assay

Ru complex effects on cell proliferation were determined via an MTT assay based on previous research with modifications [32]. The cell density seeded in the 96-well plates was 2 × 10^4^ cells/mL. A microplate spectrophotometer (VERSA max, Molecular Devices, CA, USA) was used to measure the color intensity of the formazan solution stranding at 575 nm to determine the cell viability [6].

### 3.3. Cell Migration and Invasion

To assess cell migration, HepG2 cells (1 × 10^6^ per well) were cultured in 6-well plates and allowed to form a confluent monolayer for 24 h. After the serum was starved for 4 h, the cells were scratched using pipette tips, washed with PBS, and photographed using a phase-contrast microscope (200×, Nikon TS100, Nikon, Tokyo, Japan). Fresh medium supplemented with 1% FBS was added to the wells with different Ru complex concentrations. Following incubation for 24 and 48 h, three random areas of cells were photographed. The migrated cells were subsequently quantified via manual counting, and the inhibition ratio was expressed as a percentage of the control.

The effects of Ru complexes on the invasion of HepG2 cells were assessed using a Transwell Boyden chamber (8 μm pore, Corning, Lowel, MA, USA), which was pre-coated with matrigel for 4 h at 37 °C. One hundred microliters of cell suspension (2.5 × 10^5^ cells/mL) in serum-free medium was added to the upper chamber compartment. The bottom chambers were supplemented with 500 μL of complete medium (10% FBS) and contained the indicated Ru complex concentrations. Following incubation for 24 h, the non-migrant cells from the upper face were scraped using a cotton swab. The invaded cells on the lower face were fixed with methanol, stained with Giemsa staining solution, and photographed using a phase-contrast microscope (200×, Nikon TS100). The invaded cells were quantified via manual counting, and the inhibition ratio was expressed as a percentage of the control.

### 3.4. Flow Cytometric Analysis

Following exposure to different Ru complex concentrations, the cells were washed twice with PBS and subsequently fixed with 70% ethanol at −20 °C in the dark overnight. The fixed cells were stained with propidium iodide (PI, 50.1 μg/mL) for 2 h in the dark and subsequently subjected to a Coulter Epics XL flow cytometer (Miami, FL, USA). Apoptotic cells were quantified based on the sub-G1 peak in the cell cycle distribution histogram [33].

### 3.5. Apoptotic DNA Fragmentation Assessment via TUNEL Staining Assays

Following treatments with different Ru complex concentrations, the cells cultured in confocal dishes were fixed with formaldehyde for 10 min and permeabilized with 0.1% Triton X-100 in PBS for 2 min. The cells were incubated with a 100-μL/well TUNEL reaction mixture, which contained a nucleotide mixture and terminal deoxynucleotidyl transferase (TdT), for 1 h. The cells were then stained with DAPI (1 μg/mL) for 20 min. After washing with PBS, the cells were examined using a confocal fluorescence microscope (LSM 510 META, Carl Zeiss, Jena, Germany) [34].

### 3.6. Caspase Activation by Ru Complexes

The enzymatic activities of caspase-3, -8, and -9 in Ru complex-treated cells were determined using a fluorometric method with specific caspase substrates (Ac-DEVD-AMC for caspase-3, Ac-IETD-AMC for caspase-8, and Ac-LEHD-AMC for caspase-9 purchased from Enzo Life Sciences (Plymouth Meeting, PA, USA), and the excitation and emission wavelengths were set at 380 and 440 nm, respectively [35].

### 3.7. Western Blotting

Following Ru complex treatments, the cells were harvested, collected as cell pellets, and lysed in RIPA cell lysis buffer on ice for 1 h. The cell lysate protein concentrations were determined using a BCA assay kit (Sigma-Aldrich) as previously described [36]. Equal proteins from each treatment were separated on a 12% SDS denaturing polyacrylamide gel and electrophoretically transferred to PVDF membranes. After blocking with 5% non-fat milk, the membranes were incubated with primary antibodies (1:1000; Cell Signaling Technology, Inc. (Beverly, MA, USA)) at 4 °C overnight. After washing with TBS solution, the PVDF membranes were incubated with corresponding secondary antibodies and visualized using a Pierce ECL Western blotting substrate.

### 3.8. ROS Generation Assessment

Intracellular ROS generation following Ru complex exposure was determined via a 2′,7′-dichlorodihydrofluorescein diacetate (DCFH-DA) fluorometric assay [37]. Briefly, HepG2 cells were seeded in 96-well microplates at 6 × 10^4^ cells/well for 24 h. The cells were subsequently incubated with different treatments for different lengths of exposure. Following incubation, the treated cells were incubated with 10 μM of DHE at 37 °C for 30 min. The medium was aspirated, and the cells were washed two times using PBS. Prior to ROS measurement, 100 μL of PBS was added to each well. The ROS in the samples were then immediately determined. ROS generation was assessed via fluorescence intensity with settings of 485 and 525 nm for the excitation and emission wavelengths, respectively. The change in intracellular ROS levels of each group was determined by calculating Δ*F* = (*F* − *F*0)/*F*0, where *F* represents the fluorescence read at each time point and *F*0 represents the control fluorescence.

### 3.9. Immunofluorescence Analysis of Protein Expression in Cells

Immunofluorescence imaging of Nrf2 in the treated cells was analyzed via immunofluorescence. Following Ru complex treatments, the cells were washed with PBS and fixed with 3.7% formaldehyde in PBS. After several rinses with PBS, the cells were permeabilized with 0.2% Triton X-100 for 5 min. The permeabilized cells were subsequently blocked with 0.1% BSA and incubated overnight with p-p53 primary antibody at 4 °C. The cells were incubated with Alexa-488 labeled anti-rabbit IgG antibody (1:250) for 1 h at room temperature. The cell nuclei were stained with Hochst 33342 for 20 min. Finally, the slides were analyzed with a confocal fluorescence microscope (LSM 510 META).

### 3.10. Tumor Xenograft in Nude Mice

HepG2 cells (5 × 10^6^) suspended in PBS were subcutaneously injected into the right lower hind flank of six-week-old male nude mice. The mice were then randomly assigned to three groups (*n* = 10 per group). After ten days, Ru complexes dissolved in solution (DMF_v_:Tween-80_v_:saline_v_ = 10:2:88) were administered (2.5 and 5.0 mg/kg body weight on alternating days, intraperitoneal injection) for 20 days. The control group comprised mice administered an equal volume of the vehicle (saline). The body weights and tumor volumes were assessed every 2 days. At the study endpoint, the tumor xenografts were collected and weighed. The tumor dimensions were determined using calipers. The tumor volume was calculated as follows: volume = *l* × *w*^2^/2, where *l* represents the maximal length, and *w* represents the width. All animal studies were approved by the Animal Experimentation Ethics Committee of Southern Medical University.

### 3.11. Statistical Analysis

All experiments were performed at minimum in triplicate. The data are presented as means ± standard errors. Differences between two groups were analyzed via two-tailed Student’s tests, whereas differences among three or more groups were analyzed via one-way analysis of variance (ANOVA) multiple comparisons. * *p* < 0.05 and ** *p* < 0.01 were considered statistically significant.

## 4. Conclusions

In summary, the current study elucidated the *in vitro* and *in vivo* anticancer efficacy and mechanisms of two Ru complexes. These findings indicate that these potent Ru complexes suppress the cell growth of the tested HCC cells through the induction of cell apoptosis via Nrf2 signaling pathway regulation. The cell apoptosis was evidenced by DNA fragmentation and nuclear condensation, which was predominately triggered via caspase family member activation. Furthermore, **Ru2** also demonstrated potent *in vivo* anticancer efficacy in a nude mouse model. Taken together, these findings illustrate the therapeutic potential of Ru complexes against HCC via Nrf2 pathway regulation.
